# The Role of Ancestral Duplicated Genes in Adaptation to Growth on Lactate, a Non-Fermentable Carbon Source for the Yeast *Saccharomyces cerevisiae*

**DOI:** 10.3390/ijms222212293

**Published:** 2021-11-14

**Authors:** Florian Mattenberger, Mario A. Fares, Christina Toft, Beatriz Sabater-Muñoz

**Affiliations:** 1 Integrative and Systems Biology Group, Department of Abiotic Stress, Institute for Cellular and Molecular Biology of Plants (IBMCP) from the Spanish National Research Council (CSIC), Polytechnic University of Valencia (UPV), 46022 Valencia, Spain; florian.mattenberger.1@ulaval.ca; 2 Department of Genetics, Smurfit Institute of Genetics, University of Dublin, Trinity College, Dublin 2, Ireland; 3 Panomics and Evolutionary Systems Microbiology Group, Program Systems Biology of Molecular Interactions and Regulation, Institute for Integrative Systems Biology (I2SysBio), Joint Institute of the Spanish National Research Council (CSIC), Universidad de Valencia (UV), 46980 Paterna, Spain

**Keywords:** whole-genome duplicates, small-scale duplicates, phenotypic response, metabolic distance, acidic stress, reactive oxygen response, heat-shock proteins

## Abstract

The cell central metabolism has been shaped throughout evolutionary times when facing challenges from the availability of resources. In the budding yeast, *Saccharomyces cerevisiae*, a set of duplicated genes originating from an ancestral whole-genome and several coetaneous small-scale duplication events drive energy transfer through glucose metabolism as the main carbon source either by fermentation or respiration. These duplicates (~a third of the genome) have been dated back to approximately 100 MY, allowing for enough evolutionary time to diverge in both sequence and function. Gene duplication has been proposed as a molecular mechanism of biological innovation, maintaining balance between mutational robustness and evolvability of the system. However, some questions concerning the molecular mechanisms behind duplicated genes transcriptional plasticity and functional divergence remain unresolved. In this work we challenged *S. cerevisiae* to the use of lactic acid/lactate as the sole carbon source and performed a small adaptive laboratory evolution to this non-fermentative carbon source, determining phenotypic and transcriptomic changes. We observed growth adaptation to acidic stress, by reduction of growth rate and increase in biomass production, while the transcriptomic response was mainly driven by repression of the whole-genome duplicates, those implied in glycolysis and overexpression of ROS response. The contribution of several duplicated pairs to this carbon source switch and acidic stress is also discussed.

## 1. Introduction

*Saccharomyces cerevisiae*, the budding yeast, uses glucose as its main carbon source to obtain energy, as with many other organisms. Similar to numerous other yeast species, it can alternate between fermentative and oxidative metabolism, depending on the availability of glucose and oxygen [1]. In addition, this species is able to use alternative carbon sources by adapting growth profiles [2]. This growth adaptation/versatility has been of high interest for biotechnology, from the fuel industry to biomedical/pharmaceutical industries, driving the interest in metabolism adaptation through genome modifications (i.e., adding new transporters or enzymes from closely related species or bacterial species of interest) (reviewed in [3]). This interest also relies on the tolerance to industrial bioprocess conditions, on which the yeast should be able to respond to environmental challenges or stress factors (i.e., acidic conditions, ethanol concentration, low or high temperatures) including increased concentrations of the selected compound. Under these bioprocessing conditions, yeasts might not be alone; they interact with other cells and sometimes with other species, increasing selective forces in such environments and intrinsically modifying stress responses. Indeed, it has been recently postulated that stress-free microbes lack vitality (reduced growth ability, reproduction, and longevity), indicating that any organism can cope with stressing conditions through multiple layers of response that is being driven and shaped by its genome [4] (and references within). How then does the yeast respond to these environmental challenges?

Around 100 MYA, a hybridization event took place between two different ancestral yeast species at the same time as the appearance of the first flowering plants, which established the new nectar (sugars) environments [5,6,7]. The resulting hybrids restored its fertility, with its genome reshaped by this whole-genome duplication (WGD) event followed by genome reduction and by numerous small-scale duplication (SSD) events, giving rise to glucose alcoholic fermentation metabolism and glucose-mediated repression (Crabtree effect) to cope with the new energy source environment, the hexose-sugar based nectars [8,9]. *Saccharomyces cerevisiae* is one of the descendants of this ancestral hybridization event, currently maintaining a third of its genome in duplicate [5,6,10]. In more recent works, it has been demonstrated that hybridization events among wine yeasts are produced mainly through rare mating types, giving rise to new hybrids with differential duplicated chromosome retention linked to adaptive functional innovation [11,12,13]. These works support the prevalence of duplicates and innovative potential as proposed more than fifty years ago by Ohno [14]. Since then, the molecular mechanisms behind this innovative potential and duplicate retention have become the target of many other researchers, involving the study of transcriptomic divergence between copies, functional specialization, adaptive potential, and evolvability of duplicates, among other parameters in all life kingdoms [15,16,17,18,19,20,21,22,23]. Some of these latest works show a relationship between duplicate transcriptional divergence and transcriptional plasticity in response to environmental challenges, which may link the evolvability of duplicates and their functional innovative potential in yeasts [23,24,25,26]. In recent years, we have been able to demonstrate the role of these ancestral duplicates in the stress response when challenging the budding yeast to alternative non-fermentable carbon sources, such as glycerol and ethanol [26,27]. Allowing us to unveil a common mechanism for both types of stresses, as both molecules (glycerol and ethanol) not only impose a change in carbon source, but they also induce other stresses such as osmotic pressure under low water activity and abiotic factors. We wanted to ascertain whether there is a common signature for all kinds of stresses that affect yeast growth, and how ancestral duplicates are involved in such signatures.

Skoneczny and Skoneczna [28] reviewed the response mechanisms to chemical and physical stresses in several species of yeast, including *S. cerevisiae,* and their response to acidic conditions [28]. At low extracellular pH, the protonated form can cross the cell membrane by simple or facilitated diffusion, whereas at the cytosol, the physiological pH (~7.0) induces carboxylic acid dissociation into acid anions inducing acidic stress. This acidic stress can be counteracted by the organism stress response by activating H+ -ATPases or multidrug transporters that pump out the acid anions [28,29,30,31]. However, little attention has been paid to the reactive oxygen species (ROS) response, the involvement of chaperones, or to the implication of duplicated genes, when cells were faced with the usage of these non-fermentable molecules as the energy source.

The main aim of this work was to unveil the implication of duplicated genes in the use of lactic acid as the sole carbon source while also facing acidic stress in the yeast *S. cerevisiae*. From the phenotypic point of view, we found that the growth rate was negatively affected, whereas biomass production was increased after a short adaptive process. This phenotypic change correlates with expression changes to the use of lactic acid/lactate as the non-fermentable carbon source, being the transcriptomic response mainly driven by ancestral duplicates, especially by WGDs. This transcriptomic response induced a cellular response that explains the observed phenotypic changes, and highlights the importance of other stress responses, such as ROS response similar to those observed with osmotic or ethanol stresses, or the chaperone implication at the adaptive process. These aspects are discussed in this paper.

## 2. Results

### 2.1. Lactic Acid as a Non-Fermentable Carbon Source Affects S. cerevisiae Growth Parameters

**Figure 1:**
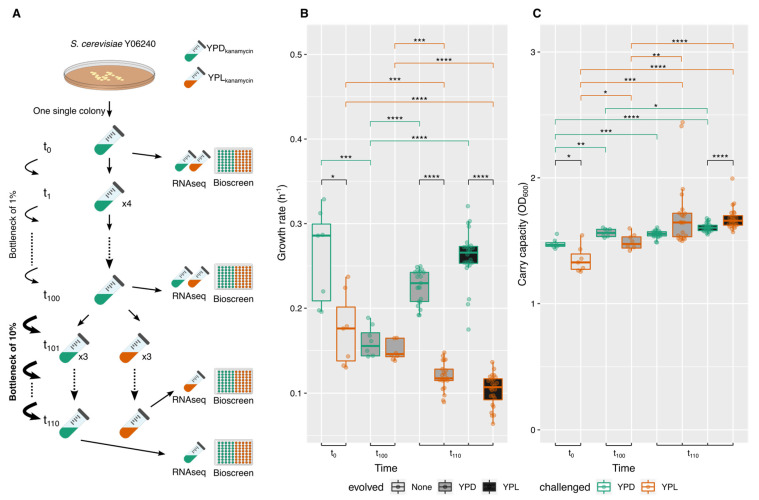
*Saccharomyces cerevisiae* laboratory evolution under diversification and adaptation to YPL and phenotypic effects on growing parameters. (**A**) Scheme of experimental evolution setup, with 1% bottlenecks during the first 100 passages (population diversification stage), and adaptive laboratory evolution to YPL or YPD under 10% population bottlenecks for additional 10 passages; (**B**) Growth rate (r, in h^−1^, as inferred from Growthcurver) for each time point and lines series, with the indication of the evolved and challenged media, note that the t_0_ populations were not evolved, but reared in YPD medium; (**C**) Carrying capacity (k), as maximum OD_600_ (as inferred from Growthcurver) for each time point and lines series, following the same criteria as in (**B**). Statistical differences among means were determined with *, **, *** or **** when the probabilities are *p*  <  0.05, *p*  <  0.005, *p*  <  0.001, and *p* < 0.0001, respectively, using a Wilcoxon rank test.

To test the ability of the yeast to overcome a challenge with a non-fermentable carbon source as lactic acid/lactate, the yeast *Saccharomyces cerevisiae* strain Y06240 was evolved first through a daily 1% bottlenecking in YPD for 100 passages (~6.6 generations per passage; ~660 generations). One of the evolved populations was then selected and subjected to ALE (adaptive laboratory evolution) to YPL (stressing media with 1% yeast extract, 2% peptone, and 3% lactic acid as the sole carbon source, at pH 5.5) by daily passages of 10% of the population for another 10 passages (~3.3 generations per passage; ~33 generations on adaptive evolution), also maintaining a control line in YPD under the same conditions; both lines were in triplicate. After evolution, cells from the fossil records at different time points were recovered and subjected to phenotypic characterization through determination of growing parameters with Bioscreen c, and transcriptomic profiling by RNAseq (Figure 1A; see Material and Methods Section for further details).

We determined the maximum growth rate (r, Figure 1B, and Supplementary Materials Figure S1A) and carrying capacity (k, Figure 1C and Supplementary Materials Figure S1B) for each population in the evolved and challenged media (YPD and YPL), from OD_600_ time course log_2_-adjusted model as implemented in Growthcurver [32]. Both parameters were used as a measurement of the populations’ fitness in each medium. The starting yeast population (t_0_) showed a sigmoidal OD_600_ curve with a maximum growth rate (r ± s.d.) of 0.261 ± 0.055 h^−1^ in YPD and of 0.175 ± 0.043 h^−1^ in YPL, being significantly lower in the second as expected due to the acidic stress (Wilcoxon rank test, *p*-value = 0.0175; Figure 1B and Supplementary Materials Figure S1A). The evolved population t_100_ also showed a sigmoidal curve in YPD, but with a lower growth rate of 0.195 ± 0.049 h^−1^ (Wilcoxon rank test, *p*-value = 5.83 × 10^−4^), and when challenged to YPL growth rate, was even lower (r = 0.141 ± 0.030 h^−1^; Wilcoxon rank test, *p*-value = 5.349 × 10^−2^), not observing statistical differences among media at this time point (Wilcoxon rank test, *p*-value = 0.165). At t_110_, the control population (Dat_110_, being evolved in YPD) showed also sigmoidal curve in YPD recovering growth rate (r = 0.257 ± 0.062 h^−1^) compared to t_0_ (Wilcoxon rank test, *p*-value = 5.827 × 10^−4^), but showed a reduced growth rate when challenged to YPL (r = 0.120 ± 0.016 h^−1^; Wilcoxon rank test, *p*-value = 4.15 × 10^−4^). Whereas YPL-adapted t_110_ population (Lat_110_) had reduced growth rate in YPL (r = 0.104 ± 0.019 h^−1^; Wilcoxon rank test, *p*-value = 3.04 × 10^−6^) compared to t_100_ challenged to YPL, and showed a higher response when challenged to the ancestral medium, YPD (r = 0.262 ± 0.032 h^−1^; Wilcoxon rank test, *p*-value = 3.04 × 10^−6^; Figure 1B).

Interestingly, we observed that carrying capacity (k) showed a positive trend that indicates phenotypic adaptation to the new environment (Figure 1C). At t_0_, carrying capacity was 1.477 ± 0.039 in YPD and 1.351 ± 0.105 in YPL, being significantly different (Wilcoxon rank test *p*-value = 1.74 × 10^−2^). At t_100_, carrying capacity was 1.549 ± 0.098 in YPD and 1.576 ± 0.364 in YPL, with no significant differences between them (Wilcoxon rank test *p*-value = 0.0728). At t_110_, populations showed bigger differences with starting population (Figure 1C and Supplementary Materials Figure S1B). Control population (Dat_110_) had a k of 1.529 ± 0.050 in YPD and a k of 1.692 ± 0.222 in YPL being significantly different from t_0_ (Wilcoxon rank test, *p*-value = 4.1610 × 10^−4^ in YPD; *p*-value = 1.11 × 10^−4^ in YPL). The YPL-adapted t_110_ population (Lat_110_) had k of 1.677 ± 0.088 in YPL and a k of 1.606 ± 0.034 in YPD, being significantly different (Wilcoxon rank test, *p*-value = 8.702 × 10^−5^) among media and respect to t_0_ population (Wilcoxon rank test *p*-value = 7.61 × 10^−7^ in YPL; *p*-value = 1.52 × 10^−6^ in YPD; Figure 1C).

### 2.2. Transcriptional Response of S. cerevisiae to Lactic Acid/Lactate as Non-Fermentable Carbon Source

**Figure 2:**
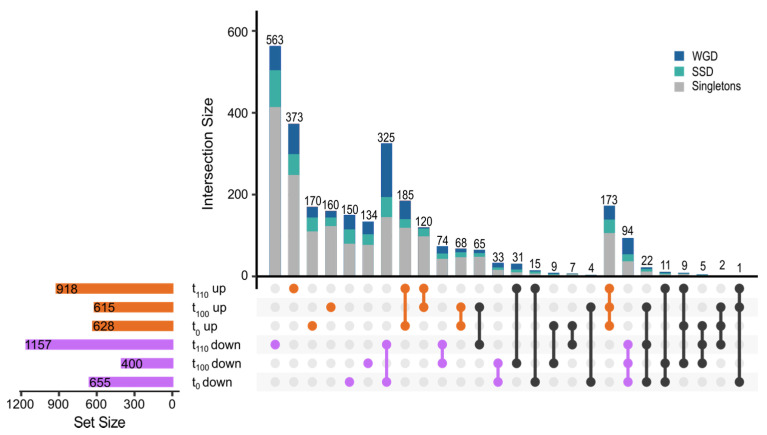
Comparison of de-regulated genes in response to YPL in the yeast *Saccharomyces cerevisiae* under diversification or adaptive experimental evolution. The UpSet graph shows the number of genes (singletons, WGDs or SSDs) in each category, shared or discordant between each data set. Up-regulated genes shown in orange, down-regulated in violet, and the discordant gene shown in black at different time-points. Graph drawn by the R package UpSetR [33].

The genetic transcriptional response of the yeast cells to a non-fermentable carbon source, as the lactic acid/lactate, was determined by RNA sequencing with comparison between time points and conditions (Figure 1A). The transcripts were mapped to a total of 6692 genes. At t_0_, when challenged to YPL (compared to YPD), a deregulation of 1283 genes (FDR < 0.005 and |log_2_FC| > 1) was observed, being almost equally distributed among up-regulated (N = 628; log_2_FC > 1), and down-regulated genes (N = 655; log_2_FC < −1; Exact binomial test: *p*-value = 0.47; Figure 2). The evolved population t_100_ challenged with YPL, showed 1015 deregulated genes, not being evenly distributed between up- (N = 615) and down-regulated genes (N = 400; Exact binomial test: *p*-value = 1.59 × 10^−^^11^; Figure 2). When comparing transcriptomic response to YPL between t_100_ and t_0_, only 431 of the de-regulated genes were also altered at t_0_, with 243 up-regulated genes at t_0_ (Figure 2). This is significantly higher than the number of down-regulated genes at both populations (N = 138; Fisher’s exact test: odds ratio = 1.83, *p*-value = 3.49 × 10^−^^7^).

Indeed, to point out the high reliability between the transcriptomic data obtained here, we investigated how many genes were up-regulated at t_100_ but down-regulated at t_0_, observing only four genes, whereas nine genes were observed in the opposite direction, being down-regulated at t_100_ after being up-regulated at t_0_. This indicates that in general, the regulation sense is kept under our evolutionary experiment, meaning that up-regulated genes at t_0_ are also up-regulated at t_100_ and that down-regulated genes at t_0_ are also down-regulated at t_100_ (Figure 2).

To understand which transcriptomic changes drive the adaptation process, we analysed the transcriptome of the population t_110_, which had been grown for 10 passages in YPL, having lactic acid/lactate as the sole carbon source. In this early adaptation, 2075 genes altered their expression profile when compared to the growth in the YPD control media, showing a huge cellular reprogramming. Contrary to what was observed in the t_0_ and t_100_ populations, this adapted population showed 1157 down-regulated genes, being significantly higher than the number of up-regulated genes (N = 918; Exact binomial test: *p*-value = 1.69 × 10^−^^7^).

Comparing the altered genes at t_0_, t_100_, and t_110_, we identified a core set of 317 transcriptionally altered genes, responding to lactic acid/lactate stress (Figure 2), with 267 showing the same expression profile. More specifically, 94 genes were down-regulated and 173 genes were up-regulated at all time points. Interestingly, only 181 of the 2803 responding genes showed a different expression profile (Figure 2), 1072 genes retained the same profile and 1550 genes were profile and point specific.

### 2.3. Many Cellular Processes Are Altered When S. cerevisiae Is Challenged with Lactic Acid/Lactate

**Figure 3:**
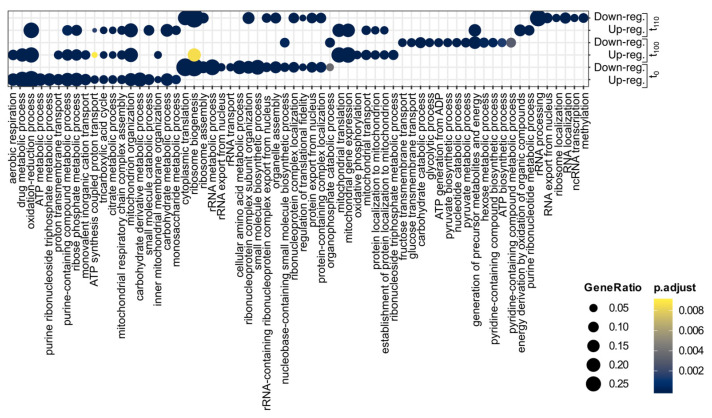
Gene ontology (GO) term enrichment analysis of deregulated genes in response to YPL in the yeast *Saccharomyces cerevisiae* under diversification or adaptive experimental evolution. GO enrichment is split for tested populations either up- or down-regulated.

To shed light on how the yeast changed its cellular response when the environment suddenly changes the carbon source, we analysed what cellular processes were altered in the different populations according to the Gene Ontology terms (GO) using the R package clusterProfiler [34]. At t_0_ up-regulated genes were enriched in functional categories, including “aerobic respiration”, “drug metabolic process”, “oxidation-reduction processes” and “ATP metabolic process”, whereas fundamental cell processes were down-regulated, including “cytoplasmic translation”, “ribosome biogenesis”, “ribosome assembly”, “rRNA metabolic process”, and “RNA transport” (Figure 3).

For the population t_100_, we observed a large overlap with t_0_ for up-regulated genes, including “aerobic respiration”, “drug metabolic process” or “oxidation-reduction processes”, and found some functional groups related to mitochondria as “mitochondrial translation”, “mitochondrial gene expression” and “oxidative phosphorylation” that were down-regulated in t_0_ (Figure 3), whereas t_100_ down-regulated gene GO enrichment included “organophosphate catabolic process”, “fructose transmembrane transport”, “glucose transmembrane transport”, and “carbohydrate catabolic process” that were almost exclusive for this time point (Figure 3).

In the population t_110_, up-regulated genes were enriched for many cellular processes shared with populations, t_0_ and t_100_, like “oxidation-reduction process”, ”purine-containing compound metabolic process” or “mitochondrion organization”; having three specific functional categories: “energy derivation by oxidation of organic compounds”, “generation of precursor metabolites and energy” and “purine ribonucleotide metabolic process” (Figure 3), whereas down-regulated gene enrichment was partially shared with t_0_, including “cytoplasmic translation”, ribosome biogenesis” or “ribosome assembly”; also showing a specific signature that included “rRNA processing”, “RNA export from nucleus”, and “ribosome localization” (Figure 3).

### 2.4. The Implication of Duplicated Genes in the Transcriptional Response of S. cerevisiae to Lactic Acid/Lactate

**Figure 4:**
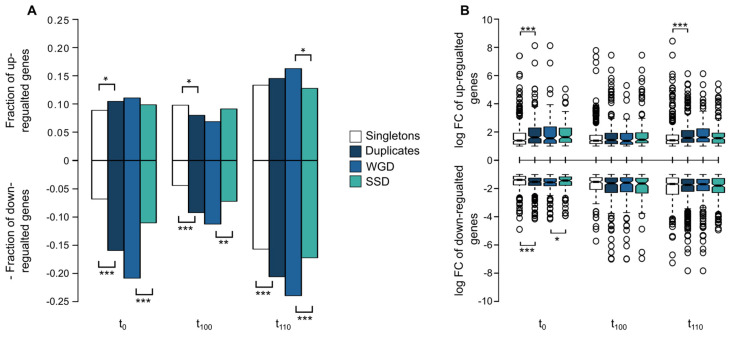
Transcriptional response to lactic acid/lactate in comparison to the response to glucose as the sole carbon source. (**A**) Distribution of fractions of deregulated genes, depending on their classification as singletons or duplicates, and within duplicates, depending on their origin, WGDs (whole-genome duplicates) or SSDs (small-scale duplicates); (**B**) Distribution of expression level as the logarithm of fold-change (log_2_FC) for singletons and duplicates, with duplicates also being divided into WGDs and SSDs. Statistical differences among means were determined with *, ** or ***, corresponding to the probabilities *p*  <  0.05, *p*  <  0.005, and *p* < 0.001, respectively, using a Wilcoxon rank test.

As indicated previously, the budding yeast keep around a third of its genome in duplicate, as a result of the ancestral whole-genome duplication, genome rearrangement and reduction, and coetaneous small-scale duplication events that took place ~100 MYA. Thus, these ancestral duplicates can be studied depending on the molecular mechanism they originate from, being divided into WGDs (whole-genome duplicates) and SSDs (small scale duplicates) [5,6,21,22,35]. For this study, we used a starting set of 1101 duplicated pairs split into 548 WGD and 553 SSD pairs, to analyse their contribution to the transcriptional and cellular response observed to lactic acid/lactate as the sole carbon source. Of this data set, we were able to identify expression data from 1094 WGDs and 1097 SSDs with a differential response at each time point (Figure 4A).

For the population t_0_, of the 1283 deregulated genes 578 were duplicated genes, observing a significantly larger fraction than expected (Exact binomial test: *p*-value < 2.2 × 10^−16^). Considering the distribution of those duplicates between the mechanisms that give rise, WGD or SSD, we observed that WGDs (N = 349) contribute significantly to the transcriptional response than expected by chance (exact binomial test: *p*-value = 5.48 × 10^−7^), whereas SSDs (N = 229) did not (Figure 4A). Out of the up-regulated 229 duplicates, we observed no statistical difference between the duplication method, WGDs (N = 121) and SSDs (N = 108; Fisher’s exact test: odds ratio F = 1.123, *p*-value = 0.406). Whereas when considering down-regulated duplicates, we observed roughly double as many WGDs (N = 228) compared to SSDs (N = 121; Fisher’s exact test: odds ratio F = 1.889, *p*-value = 9.65 × 10^−8^; Figure 4A).

For the evolved population t_100_, 377 of all deregulated genes were duplicates, a proportion higher than expected by chance (Exact binomial test: *p*-value = 3.23 × 10^−3^), as observed in t_0_, with equal contribution of WGDs (N = 198) and SSDs (N = 179; Exact binomial test: *p*-value = 0.328). However, in contradiction to what was observed at t_0_, the deregulated duplicates were not equally distributed between up-regulated (N = 175) and down-regulated duplicates (N = 202; Fisher’s exact test: odds ratio = 1.542, *p*-value = 2.383 × 10^−6^). Analysing the distribution of WGDs and SSDs for up- and down-regulated genes, we observed significantly more down-regulated WGDs than SSDs (N_WGDs_ = 123, N_SSDs_ = 79; Fisher’s exact test: odds ratio = 1.63, *p*-value = 1.13 × 10^−3^), with no difference observed between the two duplication methods in up-regulated genes (N_WGDs_ = 75, N_SSDs_ = 100; Fisher’s exact test: odds ratio = 0.734, *p*-value = 0.0584; Figure 4A).

For the population, t_110_ adapted to lactic acid/lactate, 769 of the duplicated genes altered their expression, being significantly higher than expected (Exact binomial test: *p*-value = 3.428 × 10^−5^), with the greatest contribution coming from the WGDs (N_WGD_ = 440, N_SSD_ = 329; Exact binomial test: *p*-value = 7.09 × 10^−5^). As observed in both t_0_ and t_100_, we again had a higher proportion of duplicated genes being down-regulated (N = 451) compared to singletons (N = 706; Fisher’s exact test: Odds ratio = 1.312, *p*-value = 3.92 × 10^−5^). Furthermore, WGDs (N = 262) were again overrepresented in the down-regulated duplicates compared to SSDs (N = 189; Fisher’s exact test: odds ratio F= 1.390 *p*-value = 1.16 × 10^−3^). This trend was also observed in up-regulated (N_WGD_ = 178, N_SSD_ = 140; Fisher’s exact test: odds ratio F = 1.27, *p*-value = 0.0476; Figure 4A).

Previously we determined that expression level (fold-change, FC) also contribute to duplicates preservation and to their functional innovation through response to different stresses [24]. In this case, we observed at t_0_ that duplicated genes exhibited a higher absolute log fold-change (median = 1.536) than singletons (median = 1.388; Wilcoxon test: *p*-value = 4.58 × 10^−7^) of the de-regulated genes. Furthermore, this was also the case when separating out into up- and down-regulated genes (Figure 4B). Considering the origin of those duplicates, down-regulated WGDs (median = −1.561) were more down-regulated than SSDs (median = −1.431; Wilcox test: *p*-value = 0.045; Figure 4B) whereas no differences among duplicate types were observed in up-regulated genes (median_WGD_ = 1.549, median_SSD_ = 1.631; Wilcox test: *p*-value = 0.818). At population t_100_ we did not observe any difference between expression levels of altered genes, regardless of the comparison carried out (Figure 4B). Whereas at population t_110_, one significant difference with up-regulated duplicates showed a significantly higher expression fold-change (median = 1.658) than singletons (median = 1.538; Wilcoxon test: *p*-value = 2.448 × 10^−4^), with no difference detected for this time-point (Figure 4B).

### 2.5. The Cellular Re-Programming in Response to Lactic Acid/Lactate Is Driven through Duplicated Genes

We identified the duplicated genes that were altered at the core gene set, hence those that were altered in all populations (t_0_, t_100_, and t_110_). We found that 148 duplicated genes belong to the core category, with 124 having the same expression profile at all three populations (N_up_ = 67 and N_down_ = 57). This core set gene showed enrichment of down-regulated duplicates (Fisher’s exact test: odds rate F = 3.164, *p*-value = 5.0 × 10^−8^; Figure 2) but the up-regulated genes were distributed among duplicates and singletons as would be expected (Fisher’s exact test: odds rate F = 1.298, *p*-value = 0.1015). When investigating what functional categories were affected, we observed that altered core genes were enriched mainly for functions involved in precursor metabolites and energy, such as “oxidation-reduction process”, “generation of precursor metabolites and energy”, “aerobic respiration” and “mitochondrial respiratory chain complex assembly”.

### 2.6. Chaperones, Heat Shock Proteins and Stress-Related Proteins Responding to Lactic Acid/Lactate as Carbon Source

Among the lactate quick response genes (those up-regulated at t_0_ and t_100_) we found: Gre1 (YPL223C) a WGDs responsive to stress; Fyv5 (YCL058C) a gene *de novo* originated from non-coding sequence involved in ion homeostasis and required for survival after exposure to killer toxins; and Fyv4 (YHR059W) a protein of unknown function also required for survival to killer toxins.

Among the highly expressed genes responding to acute lactate exposure (Lat_110_ population) we found several heat shock proteins: Hsp26 (YBR072W; SSD), Hsp30 (YCR021C), Hsp42 (YDR171W), Hsp78 (YDR258C), Hsp82 (YPL240C; WGD) Sse2 (YBR169C), Ssa4 (YER135C), Hsp104 (YLL026W; SSD), Ssc1 (YJR045C, HSP70 family ATPase, WGD); chaperone Apj1 (YNL077W; SSD); co-chaperones Mdj1 (YFL088W) and Sis1 (YNL007C), all involved in protein folding/refolding under heat-shock and stressing conditions (including lactic acidosis of the environment), some of them with implications on DNA replication stress. Other proteins involved in DNA replication stress, oxidative stress or stress-response elements were Ctr1 (YPR124W), Gph3 (YPR160W), Gsy2 (YLR258W; WGD), Tma10 (YLR327C; WGD), Pgm2 (YMR105C; WGD), Opi10 (YOL032W), Gad1 (YMR250W; SSD), Fes1 (YBR101C), Rgi (YER067W; WGD), Btn2 (YGR142W; WGD), Rie1 (YGR250C), Tdh1 (YJL052W), Roq1 (YJL144W) and Mrs4 (YKR052C; WGD).

### 2.7. Metabolic Evolution of YPL Adapted S. cerevisiae Populations

To determine similarities among the different populations after the experimental evolution, we measured the metabolic distance among the three populations, this being the grade of sharing of cellular GO processes to determine proximity of the populations in terms of metabolism (see Materials and Methods section). The metabolic distance (MD) was determined for all the transcriptionally altered genes (including duplicates and singletons), as well as for the up- and down-regulated genes between pairs of the t_0_, t_100,_ and t_110_ populations. As expected, we observed that the most related metabolic distance of the populations for the whole altered gene set were t_0_ and t_100_ since the population t_100_ was neutrally evolved from the population t_0_ to increase its genetic variability. The same was true for the up-regulated gene set but surprisingly not for the down-regulated gene (Table 1). When we analysed only the duplicated genes, we observed that the closest metabolic distance for all comparisons was between t_0_ and t_110_ populations.

## 3. Discussion

Yeast, like many other organisms, uses glucose as the main carbon source to obtain energy, and is able to modify its metabolism depending on the available carbon sources. The budding yeast undergoes fermentative metabolism of glucose to obtain ATP and other basic metabolic precursors from the complex YPD medium (containing 2% dextrose) having ethanol, glycerol, and lactate/lactic acid as the main by-products. These by-products can become potent control molecules that limit the development of other microorganisms by exerting different kinds of stresses or are used as alternative carbon sources to obtain energy through respiration. In this work, we determine the contribution of duplicated genes in response to the carbon source switch from glucose to lactic acid/lactate and how this transcriptional switch affected yeast growth.

### 3.1. Lactic Acid as Non-Fermentable Carbon Source Affects S. cerevisiae Growth Parameters

Lactic acid, an organic acid produced by some yeasts and lactic bacteria through sugar media fermentation, is usually produced as its conjugated anion lactate due to intracellular pH (pKa = 3.68). This weak organic acid compound has a wide range of industrial applications ranging from food preservation to pharmaceutical production to plastic production. Moreover, it is a biological stressor, by either forcing a growth arrest and a metabolic rewire of the target organism due to the lack of glucose in the medium or by acidic stress induced by the dissipation of pH gradient through the plasma membrane [28,36]. In addition, lactate has been recently classified as a signaling molecule in several human diseases, including cancer, deserving further research on its metabolism and transcriptional effects [37,38,39,40].

The presence of this weak acid in the culture media (either in small or at factory scales) induces in a first step a cell-cycle arrest, resuming cell growth after several hours. This transient growth arrest is lost when cells are pre-adapted to weak acids, such as lactic acid or acetic acid, implying a durable transcriptomic rewire [41]. In our work, we saw this growth arrest with a significant decrease of growth rate when switching carbon source from glucose to lactic acid, this decrease was also observed in the lactic acid short-adapted lines (population YPL t_110_, Figure 1B). However, as indicated, cells are able to resume cell growth after several hours, time allocated to transcriptional activity, and cell growth and reproduction, which at the end indicates an increase of the carrying capacity of the culture. Similarly, our YPL-adapted lines showed a significant increase of carrying capacity compared to the non-adapted ones (control lines), indicating successful growth resumption (see Figure 1C and Supplementary Materials Figure S1B). In other works, we demonstrated that this short adaptive experimental evolution (only 33 generations) also affected growth rate, with a significant reduction when challenged to glucose-deprived media using other non-fermentative carbon sources [26,27]. However, when using glycerol or ethanol as non-fermentative carbon sources, growth was resumed without a significant increase of biomass as observed with lactic acid/lactate (Figure 1C and Supplementary Materials Figure S1B). These results would indicate that in this case, we are not yet fixing adaptive mutations, as expected if a genotypic switch took place after the adaptive process [42,43,44,45,46,47,48,49]. But the increase of carrying capacity in the YPL adapted lines is also in agreement with an increase of TCA fluxes, as explained in [50]. Indeed, we observed phenotypic heterogeneity in the evolved cells, which decreased after adaptive evolution (see Figure 1, and Supplementary Materials Figure S1). It seems that our populations show heterogeneous phenotypes linked to transcription heterogeneity in lactic acid/lactate medium, as observed previously to adaptation to other functional trade-offs [51,52].

### 3.2. Lactic Acid as a Non-Fermentable Carbon Source Affects S. cerevisiae Transcriptomic Response

**Figure 5:**
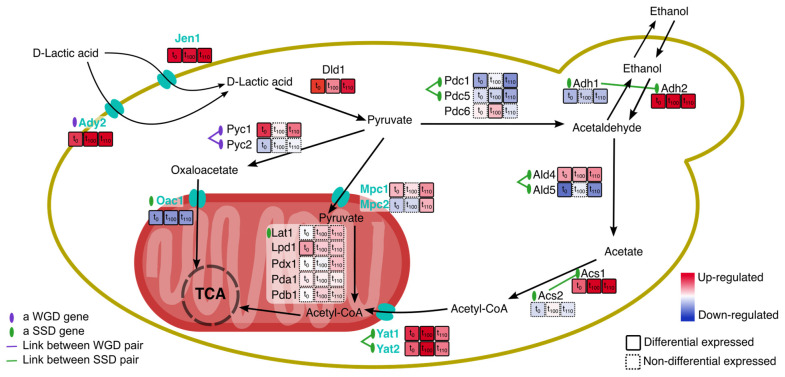
*Saccharomyces cerevisiae* Y06240 lactic acid/lactate uptake mechanism, with the indication of metabolic, rewire after experimental evolution on YPL. Lactate transporters both in periplasmic and mitochondrial membranes have been indicated in green. Some of the duplicated genes implicated in this lactate uptake and metabolism have been indicated with colors, whereas singletons are in black. Expression level (up in red and down-regulated in blue) and statistical significance (differentially expressed genes in a continuous line, and non-differentially expressed genes in dashed line) of each gene are indicated.

The improvement of lactic acid production from sugar fermentation in *S. cerevisiae* has been achieved by alcohol fermentation inhibition, by increasing methyltransferases, and by modulation of Jen1 and Ady2 monocarboxylic permeases [53,54,55]. These two transporters (repressed by glucose) mediate the export of acetate, formate, propionate, and lactate from the cytoplasm reducing the internal acidic stress and increasing the release of lactic acid. However, these two transporters also work in the reverse direction, being involved in the use of lactic acid/lactate as a carbon source by the budding yeast [54]. In this work, we forced the cell to use lactic acid/lactate as the sole carbon source, finding that both transporters Jen1 and Ady2 were overexpressed since the first challenge, and that ethanol reduction to acetaldehyde was also increased, redirecting the central metabolism to obtain energy (see Figure 5).

It has been demonstrated that under glucose limiting conditions, the budding yeast undergoes a significant transcriptional switch, with a fast response under an acute situation or with a slow response under chronic glucose limitation [56,57]. As indicated previously, the budding yeast can use several non-fermentable carbon sources that are also products of glucose metabolism, such as ethanol, glycerol, acetate, and lactate, inducing a transcriptional regulation involving overexpression of membrane transporters and activation of transcription factors for the use of these non-fermentable carbon sources [58,59]. In this work, we show that the complete replacement of glucose (dextrose) by a non-fermentable carbon source such as lactic acid/lactate in the growing medium (from YPD to YPL) drives to a complete transcriptomic re-wiring affecting central metabolism genes that would affect growing parameters (see Figure 3 and Figure 5).

Similarly, when the hybrid yeast *Zygosaccharomyces parabailii* was subjected to lactic acid supplementation with a medium rich in glucose (4%), a transcriptomic rewire was observed [60]. This species shows a weak acid stress tolerance by modulating cell wall-related genes, including the transcription factors Haa1, Aft1/Aft2 (repression of these genes to avoid lactic acid uptake), by the induction of genes involved in oxidative stress response and iron homeostasis, and by amplification by gene duplication at a small scale of formate dehydrogenase (FDH) genes, identifying such SSDs directly linked to lactic acid tolerance with differential gene expression between copies [60]. The budding yeast, *S. cerevisiae,* undergoes a different strategy (when tested under acidic stress in the presence of glucose), inducing ROS response, affecting iron homeostasis, and expending large amounts of ATP through Pdr12 ABC transporter (YPL058C) that catalyses lactic acid [41]. In our case, we observed that under media with no glucose, the budding yeast forces the entry of lactate with over-expression of transporters Jen1 and Ady2, and re-wires the central metabolism to the production of energy via pyruvate and acetate synthesis from lactate through up-regulation of a set of duplicated genes, including some related to ROS response and heat-shock family proteins (see some of the duplicates in Figure 5).

Among the responding genes to lactate in the core set (altered genes shared by all three populations), we found Idp2 (YLR174W; WGD), Cyb2 (YML054C; singleton), Mls1 (YNL117W; SSD), Acs1 (YAL054C; SSD), and Rgi2 (YIL057C; WGD) genes implied in the utilization of non-fermentable carbon sources, with affected growth parameters even under vegetative growth and decreased lifespan [58,59].

### 3.3. Ancient Duplicates Direct the Transcriptomic Response

As indicated previously, gene duplication is a major force in evolution, sourcing new genetic material and novel functions, being related to the radiation of angiosperms and developmental complexity in animals [61,62,63,64,65]. In this study, we were interested in deciphering the role of the anciently duplicated genes during the adaptation to a challenging environment, the YPL medium containing lactic acid/lactate as the sole carbon source, by analysing results of an adaptive laboratory experiment.

Experimental evolution has been a common tool in the past years to achieve insight into molecular mechanisms and cellular responses underlying adaptation [66,67,68]. As shown, we found that (Figure 2 and Figure 4) duplicates direct the transcriptomic response to an acidic environment with lactic acid/lactate as the sole carbon source after experimental evolution. Duplicates significantly alter their expression profile compared to singletons, showing also significantly higher expression fold-change than singletons (Figure 4). When studying the transcriptional response, a core set of genes was identified, with an enrichment of the WGDs. Whole-genome duplicates implied in glycolysis, glucose metabolism, and hexose catabolic process were repressed. We also observed down regulation of Sam2 (YDR502C) involved in direct lactic acid tolerance and production [54]. Up-regulated WGDs were involved in “oxidation-reduction process”, “generation of precursor metabolites and energy”, “aerobic respiration” and “mitochondrial respiratory chain complex assembly” (Figure 3). Of these pairs, we also observed a discordant trend (discordant category as in [23,24]) with opposite transcriptional response to duplicated pairs, including SSD pairs such as Acs1/Acs2, Ald4/Ald5, and Adh1/Adh2, and the WGD pair Pyc1/Pyc2 (Figure 5), and only one SSD gene pair, Yat1/Yat2, was up-regulated.

### 3.4. Lactic Acid/Lactate as the Sole Carbon Source Induces More Than a Single Stress Response

Wild-type yeasts can grow at pH values ranging from 2.5 to 8.5, with growth and fermentation kinetics not affected between pH 3.5 and 6.0. As indicated previously, lactic acid/lactate is purely a respiratory substrate for *Saccharomyces cerevisiae* that affects growth parameters by reducing growth rate and increasing biomass production. This lactate consumption increases the pH of the medium by decreasing the basal respiratory rate, mainly due to a decrease in ATP consumption linked to the maintenance of intracellular pH and reduction of the number of mitochondria per cell [69]. The intracellular pH is also a tightly controlled factor, with great differences among cell compartments (vacuole, mitochondria, nucleus, Golgi network, peroxisomes, and secretory vesicles), that alters the cellular set-up and cell physiology affecting multiple regulatory levels simultaneously [31,70]. A decrease of the internal pH will affect enzymes activity, and also by changing interaction between residues of amino acid side chains conformational stability of proteins and interactions between proteins will be affected. In addition, oxidation-reduction potential (transference of electrons and expenditure of NADPH) is also dependent on internal pH, affecting ROS response-related proteins (reviewed in [70]). Moreover, acidification of cytosol will affect other structural molecules as lipids or coupled phosphates affecting intracellular compartment membranes and DNA (reviewed in [70] and references within). Each of these steps seems to be involved in the induction of acidic, oxidative, and DNA damage stresses, which were observed in this work (see Figure 3). In addition, the conformational instability of proteins might be responsible for the observed up-regulation of a long list of heat shock proteins, chaperones, and co-chaperonins (see Section 2.6). This deserves further study to determine their implication on system robustness, as this response was only observed after chronic exposure to lactic acid/lactate. The implication of altered duplicates on such responses (to different stresses) still deserves further research, but some light has been shed on how duplicates were selected, preserved, and still able to innovate following Ohno’s dilemma [71,72].

## 4. Materials and Methods

### 4.1. Yeast Strain, Culture and Adaptive Experimental Evolution

The yeast *Saccharomyces cerevisiae* strain Y06240, a derivative of BY4741 with *msh2* gene deleted from the YKO collection (Mata; his3D1; leud2D0; met15D0; ura3D0; msh2::kanMX4), was used [21]. As previously described, a homogeneous population (t_0_) was founded by growing a single colony into 5 mL of YPD (2% (*w*/*v*) bactopeptone, 1% (*w*/*v*) yeast extract, 2% (*w*/*v*) dextrose; supplemented with 100 µg/mL kanamycin) for 24 h at 28 °C and 220 rpm, and evolved through 1% daily bottlenecks (by transferring 50 μL of culture into 5.0 mL of fresh YPD medium; ~6.6 generations per passage) for 100 passages (t_100_; ~660 generations) [26,27]. From passage 100 (t_100_), one population (a1) was selected and divided into two sets, a control set adapted to YPD (Da set), and the one challenged to YPL (La set), medium containing 3% lactic acid as sole carbon source (YPL: 3% (*v*/*v*) lactic acid, 2% (*w*/*v*) bactopeptone, 1% (*w*/*v*) yeast extract, pH 5.5; supplemented with 100 µg/mL kanamycin). Each set consisted of three biological replicates, being subjected to daily passages, with 10% population bottleneck (by transferring 500 μL of culture into 5.0 mL of fresh YPD or YPL media) for another 10 passages (~3.3 generations per passage). During the evolution, a fossil record was constructed, with glycerol stock of populations every 10 passages in the diversification experimental evolution or every 5 passages in the adaptive evolution, being preserved at −80 °C (Figure 1A).

### 4.2. Growth Characterization under Lactic Acid Use as Sole Carbon Source

Evolved yeasts at t_0_, t_100_, and t_110_ were characterized for growing ability response to carbon source, either in ancestral medium (YPD) or challenged to YPL (Figure 1A). Cells from fossil records were recovered in the corresponding growing media, by seeding 5 to 50 μL of glycerol stock into 5.0 mL of fresh medium in 50 mL conic vials. After 24 h at 28 °C and 200 rpm, cells were diluted till OD_600_ ~0.1 in 200 μL volume and growth was recorded in a Bioscreen c MBR plate reader system (Oy Growth Curves Ab Ldt., Helsinki, Finland) taking OD_600_ measurement every 15 min, with brown filter and continuous shaking at 28 °C. Each line and time points were challenged in both YPD and YPL with at least 5 technical replicates.

Growth rate (r) and carrying capacity (k) were determined from the logistic adjustment of corrected OD_600_ time data series in each well with Growthcurver package in R [32]. Means were compared among treatments and lines with Wilcoxon rank test in R [73].

### 4.3. RNAseq and Transcriptomic Profiling

Evolved yeasts at t_0_, t_100_, and t_110_ were characterized for their transcriptional response to carbon source switch or their adaptation to this acidic environment (Figure 1). As described previously, cells from fossil records were recovered in the corresponding growing media, and after reaching OD_600_ ~0.6 (~16 h at 28 °C and 200 rpm), challenged to YPD and YPL, in triplicate. Cultures were stopped at OD_600_ ~0.6 (by placing them in an ice bath), cells harvested by centrifugation and total RNA extracted using RNEasy kit (Qiagen) following manufacturer instructions. RNA quality was checked with QuBit 4, and those samples with RIN >8 were used for library construction.

Stranded RNA libraries were constructed using TruSeq stranded mRNA (Illumina) from oligo-dT captured mRNAs. Libraries were run in NextSeq 500 (Illumina) at 75 nt single read by using the High Output 75 cycles kit v2.0 (Illumina). RNA libraries were constructed and sequenced at Genomic core facility at Servicio Central de Soporte a la Investigacion Experimental (SCSIE) from University of Valencia, Spain. Raw reads were deposited at SRA NCBI database with accession numbers PRJNA321113 (t_0_ in YPD and YPL; [74]), PRJNA610243 (a1t_100_ in YPD; [26]), PRJNA610597 (a1t_100_, in YPL), PRJNA610474 (Da1t_110_, in YPD; [26]) and PRJNA610717 (La1t_110_, in YPL). Each accession number contains three libraries corresponding to the technical replicates.

Raw reads were analysed using FastQC report, cleaned with CutAdapt, and trimmed for quality and length (Pred score inferior to 20 and size less than 40 nt were discarded). Reads were aligned with Bowtie2 (up to two mismatches accepted) to the reference S288c strain genome (only CDS; assembly R64) [26]. Statistical assessment of differential gene expression was carried out with edgeR, setting false discovery rate (FDR) at <0.005, and applying BY correction for *p*-value (0.005) [75]. Comparisons were conducted between time points and media, taking into account the original transcriptomic background.

### 4.4. Identification of Duplicated Genes Involved in the Usage of Lactic Acid as Sole Carbon Source and Their Response to Acidic Stress

Differentially transcribed genes were further analysed as singletons and duplicates (homologs), duplicates were split into two, according to their mechanism of origin (WGDs or SSDs). Whole-genome duplicates (WGDs; 555 pairs) were extracted from the reconciled YGOB list v.7 (Yeast Gene Order Browser version 7 (August 2012); http://wolfe.gen.tcd.ie//ygob; [76]), while small-scale duplicates (SSDs; 560 pairs) were identified by previously carried out best reciprocal blast searches [21,22,26].

After classification of differentially expressed genes as singletons or duplicates (WGDs or SSDs), an enrichment analysis of gene ontology (GO) terms was performed using the R package cluster Profiler and *p*-value cut-off of <0.01 [34], to determine the contribution of each set in the transcriptomic response to acidic stress.

### 4.5. Metabolic Distance between Populations

The metabolic distance was calculated as previously described [27]. Briefly, lists of GO process terms enriched for transcriptionally altered genes between two populations (*i* and *j*) were compared by calculating the number of shared process terms (SP*ij*), and the number of enriched terms for transcriptionally altered genes only in one of the populations but not in the other (P*i* and P*j*), with metabolic distance (MD*ij*) calculated as:

MD*ij* = 1 − (SP*ij*/Min[P*i*, P*j*])

where Min[P*i*, P*j*] is the number of cellular processes enriched for transcriptionally altered genes for the population with the minimum number of such processes. Metabolic distance varies between 0 and 1, with smaller values indicating the closeness of populations, and a value of 1 corresponding to two complete divergent populations, as there is no overlap in the enriched process terms shared.

## 5. Conclusions

Lactic acid/lactate is an alternative non-fermentative carbon source that also induces acidic stress to yeast cells. These cells undergo cell growth arrest, with a significant reduction of growth rate, to adjust their transcription profiling. After this transcriptional rewiring, cell growth arrest is resumed thereby producing an increase of biomass (measured as carrying capacity), even after a short-term adaptive process (33 generations). The transcriptional rewire of central metabolism includes down-regulation of WGDs implied in glycolysis, and up-regulation of several WGDs and SSDs involved in transcription adjustment, in the oxidative stress response and showing the implication of several heat shock proteins and chaperones. With this work, we add a further step in determining duplicated genes’ central role in acute and chronic response to stress, ultimately with their continued involvement in the adaptation process.

## Figures and Tables

**Table 1:** Metabolic distances between evolved populations in response to lactic acid/lactate as sole carbon source.

	Altered			Up-Regulated			Down-Regulated		
	t_0_–t_100_	t_0_–t_110_	t_100_–t_110_	t_0_–t_100_	t_0_–t_110_	t_100_–t_110_	t_0_–t_100_	t_0_–t_110_	t_100_–t_110_
All	0.1914	0.3713	0.5359	0.3295	0.2897	0.2890	0.6082	0.4021	0.9278
Singletons	0.3302	0.5934	0.3585	0.3028	0.2611	0.1549	0.5000	0.4789	1.0000
Duplicates	0.4955	0.1279	0.8488	0.2466	0.4028	0.5694	0.9048	0.1684	0.9365
WGDs	0.3962	0.0132	0.9623	0.0667	0.5652	0.6522	0.8750	0.0435	0.9464
SSDs	0.4815	0.7692	0.7037	0.5641	0.5455	0.3636	0.8667	0.5111	0.9000

## Data Availability

Raw reads (fastq files) are available from the Sequence Read Archive (SRA) with accession numbers PRJNA321113 (t_0_ in YPD and YPL), PRJNA610243 (a1t_100_ in YPD), PRJNA610597 (a1t_100_, in YPL), PRJNA610474 (Da1t_110_, in YPD) and PRJNA610717 (La1t_110_, in YPL).

## Supplementary Materials

The following are available online at https://www.mdpi.com/article/10.3390/ijms222212293/s1.
