# Supplementary material for: The Role of Ancestral Duplicated Genes in Adaptation to Growth on Lactate, a Non-Fermentable Carbon Source for the Yeast Saccharomyces cerevisiae
Source: Int J Mol Sci. 2021 Nov 14;22(22):12293. doi: 10.3390/ijms222212293 (PMC8622941; doi:10.3390/ijms222212293)
Supplement: Supplementary file 1 [file ijms-22-12293-s001.zip › ijms-1453049-supplementary.pdf]

Supplementary Figure S1.

The role of ancestral duplicated genes in adaptation to growth on lactate, a non-fermentable carbon source for the yeast *Saccharomyces cerevisiae*

Florian Mattenberger [1,#,$], Mario A. Fares[1,2, ±], Christina Toft [3,#,*] and Beatriz Sabater-Munoz [1,*]

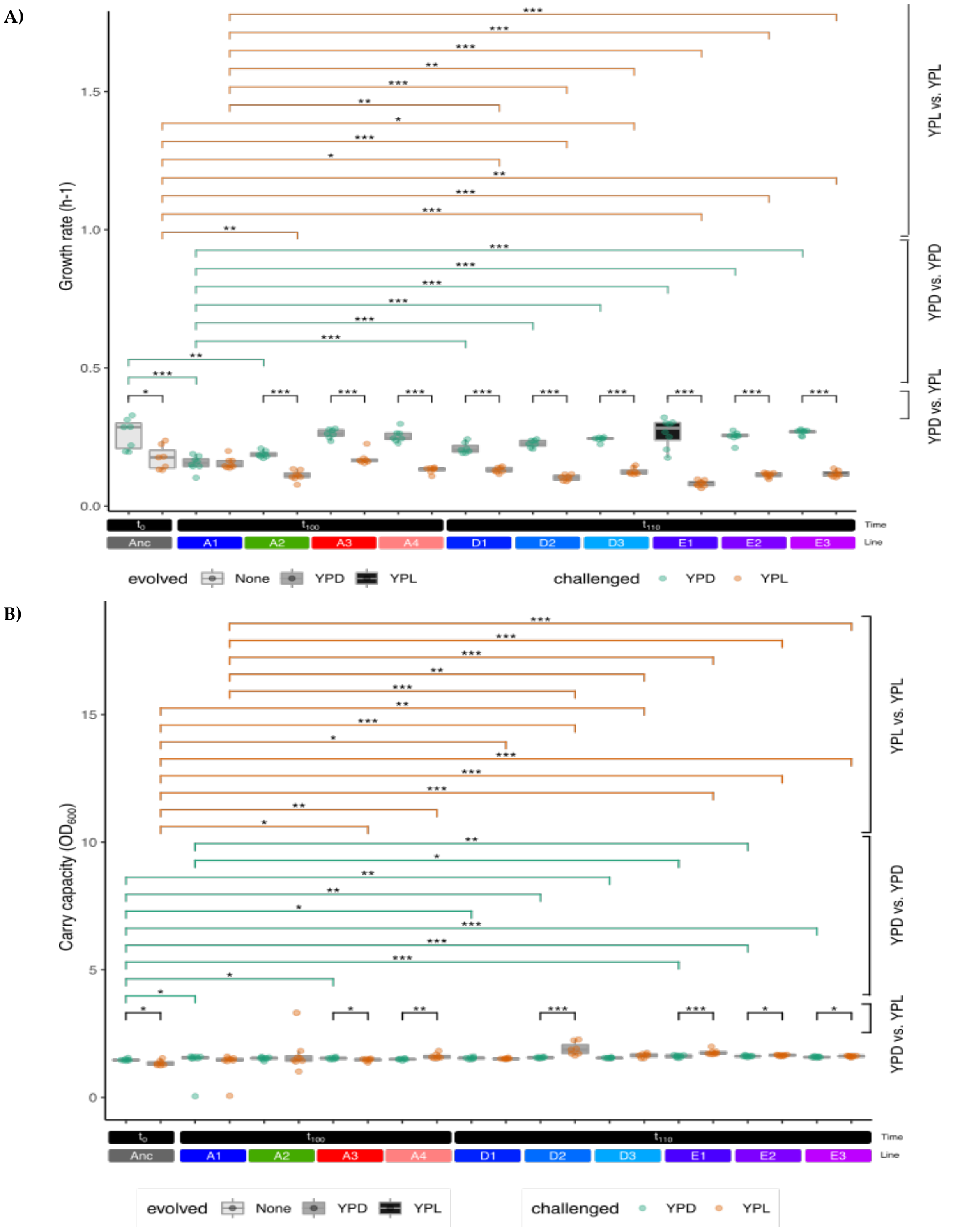

Figure S1. Growth parameters of *S. cerevisiae* Y06240 lines subjected to genetic diversification or to adaptive experimental evolution. A) Growth rate (h^{-1}) per line and time point; B) Carrying capacity (k, ODmax) per line and time point.
